# Electrochemical Investigation of Iron-Catalyzed Atom Transfer Radical Polymerization

**DOI:** 10.3390/molecules27196312

**Published:** 2022-09-24

**Authors:** Gianluca Gazzola, Sebastiano Pasinato, Marco Fantin, Niccolò Braidi, Cristina Tubaro, Christian Durante, Abdirisak Ahmed Isse

**Affiliations:** 1 Department of Chemical Sciences, University of Padova, Via Marzolo 1, 35131 Padova, Italy; 2 Department of Chemical and Geological Sciences, University of Modena and Reggio Emilia, Via Campi 103, 41125 Modena, Italy

**Keywords:** iron catalysts, *e*ATRP, methyl methacrylate, radical polymerization

## Abstract

Use of iron-based catalysts in atom transfer radical polymerization (ATRP) is very interesting because of the abundance of the metal and its biocompatibility. Although the mechanism of action is not well understood yet, iron halide salts are usually used as catalysts, often in the presence of nitrogen or phosphorous ligands (L). In this study, electrochemically mediated ATRP (*e*ATRP) of methyl methacrylate (MMA) catalyzed by FeCl_3_, both in the absence and presence of additional ligands, was investigated in dimethylformamide. The electrochemical behavior of FeCl_3_ and FeCl_3_/L was deeply investigated showing the speciation of Fe(III) and Fe(II) and the role played by added ligands. It is shown that amine ligands form stable iron complexes, whereas phosphines act as reducing agents. *e*ATRP of MMA catalyzed by FeCl_3_ was investigated in different conditions. In particular, the effects of temperature, catalyst concentration, catalyst-to-initiator ratio, halide ion excess and added ligands were investigated. In general, polymerization was moderately fast but difficult to control. Surprisingly, the best results were obtained with FeCl_3_ without any other ligand. Electrogenerated Fe(II) effectively activates the dormant chains but deactivation of the propagating radicals by Fe(III) species is less efficient, resulting in dispersity > 1.5, unless a high concentration of FeCl_3_ is used.

## 1. Introduction

Reversible deactivation radical polymerization (RDRP) techniques allow for the preparation of polymeric materials with precisely tailored architectures, low dispersity and well-preserved chain end functionality [1]. Among these methods, the most important and widely used techniques are atom transfer radical polymerization (ATRP [2,3,4]), reversible addition fragmentation chain transfer polymerization [5,6] and nitroxide-mediated polymerization [7,8]. The success of these methods relies on establishing a dynamic equilibrium between the propagating radical and a dormant species, which drastically reduces the rate of termination reactions, resulting in a remarkable increase in radical lifetime.

**Scheme 1:**
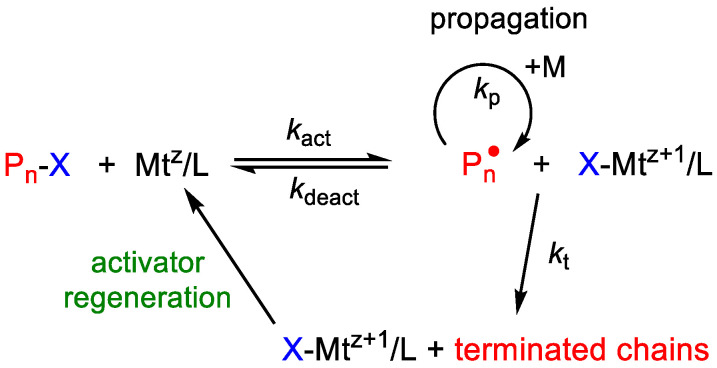
General mechanism of ATRP with a reduction loop for activator regeneration (X = Cl, Br).

ATRP uses a transition metal complex, mainly Cu with a nitrogen-based ligand [3], to establish the equilibrium between a dormant polymer chain and the corresponding active radical. A metal complex at a low oxidation state (Mt^z^/L) reacts with an alkyl halide (RX) initiator or a halogen-capped dormant polymer chain (P_n_-X) to produce a propagating radical (P_n_^●^) and the metal complex at a higher oxidation state (X-Mt^z+1^/L) with the halide ion as an additional ligand (Scheme 1). This activation step occurs according to an inner-sphere electron transfer mechanism [9,10,11]. The radical propagates for a short period before it is quenched by the deactivator complex X-Mt^z+1^/L to form a dormant species. The equilibrium constant, *K*_ATRP_ = *k*_act_/*k*_deact_, defines the equilibrium concentration of propagating radicals and, hence, plays a crucial role in polymerization kinetics and control over molecular weight distribution.

Several advanced variants of ATRP have been developed to reduce catalyst loading and improve the industrial attractiveness of the process. In these methods, the process starts with an oxygen-stable metal catalyst at a high oxidation state, while the activator catalyst—the metal at a lower oxidation state—is (re)generated in situ through various approaches [12]. Examples of these methods include activators regenerated by electron transfer (ARGET) ATRP [13,14,15] and supplemental activator and reducing agent (SARA) ATRP (also known as SET LRP) [16,17,18], which use a homogeneous reducing agent and a zero-valent metal, respectively, and methods based on external stimuli via electrochemistry [19,20], photochemistry [21,22,23] and sonochemistry [24,25]. Metal-free photo-induced ATRP is also widely used [26,27].

Although Cu is the most popular metal in ATRP, polymerizations have successfully been carried out with catalysts based on a large variety of other metals, such as Re, Fe, Ru, Os, Rh, Co, Ni and Pd [28]. Of these, iron has attracted considerable interest because it presents several advantages over copper catalysts. Indeed, iron is an extremely abundant metal in the Earth’s crust and biocompatible iron-based systems can potentially be used for biomedical applications [29,30]. Therefore, iron can be considered a very promising metal in view of industrial development of ATRP. Like Cu and other metal catalysts, the activity of Fe-based catalysts strongly depends on the nature and structure of the ligands [31]. Most Fe catalysts used in ATRP are based on iron halide salts, FeX_3_ and/or FeX_2_, in the absence or presence of added ligands, mainly based on nitrogen and phosphorous [32].

Both conventional ATRP based on Fe(II) [33,34,35] and advanced variants, such as initiators for continuous activator regeneration (ICAR) ATRP [36,37,38,39], ARGET ATRP [40,41], photo-induced ATRP [39,42,43] and electrochemically mediated ATRP (*e*ATRP) [44,45,46], have been used. Iron-based biocatalysts have also been successfully used in surface-initiated ATRP [47] as well as in solution phase [48,49], with interesting applications in biosensing [50,51,52]. *e*ATRP is particularly attractive because it is environmentally friendly, as no chemical reducing agents are used and it allows easy regulation of polymerization rate and accurate temporal control [53,54]. Unlike copper-catalyzed radical polymerization, for which *e*ATRP has been successfully applied to a wide range of monomers in various reaction media [19,20], *e*ATRP with Fe-based catalysts has rarely been investigated. The first application of electrochemistry to trigger controlled radical polymerization was reported by Amatore and co-workers who investigated the reaction of electrogenerated Fe^II^(salen) (H_2_salen = *N*,*N*’-bis(salicylidene)ethylenediamine) with some alkyl halide initiators and its application to styrene polymerization [55]. More recently, Guo et al. [44,45] and Wang et al. [46] reported *e*ATRP of methyl methacrylate (MMA) catalyzed by iron halide salts, with or without tris(4-methoxyphenyl)-phosphine as an additional ligand, respectively.

**Scheme 2:**
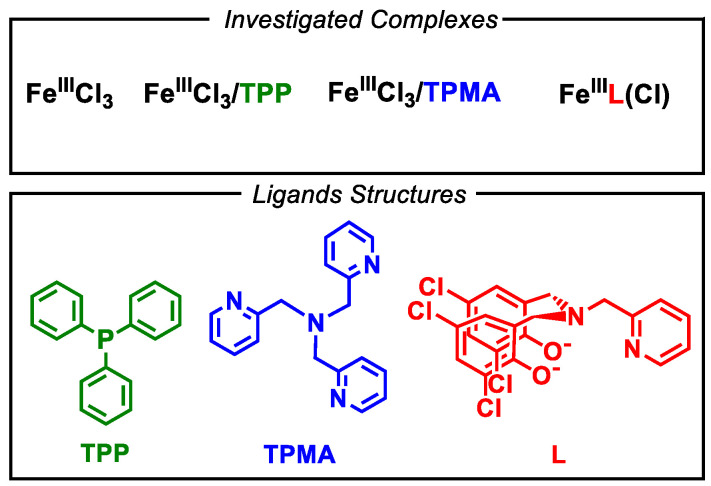
Investigated Fe complexes. TPP = triphenylphosphine; TPMA = tris(2-pyridylmethyl)amine; L = 2-pyridylamino-*N*,*N*-bis(2-methylene-4,6-dichlorophenolate).

Herein, we report a deeper investigation on the electrochemical behavior of the most common iron-based catalysts used in ATRP. The study also addresses the role of added ligands, such as chloride anions, amine–bis(phenolate), phosphines and amines (structures in Scheme 2), on *e*ATRP of MMA.

## 2. Results and Discussion

### 2.1. Cyclic Voltammetry of FeCl_3_

**Figure 1:**
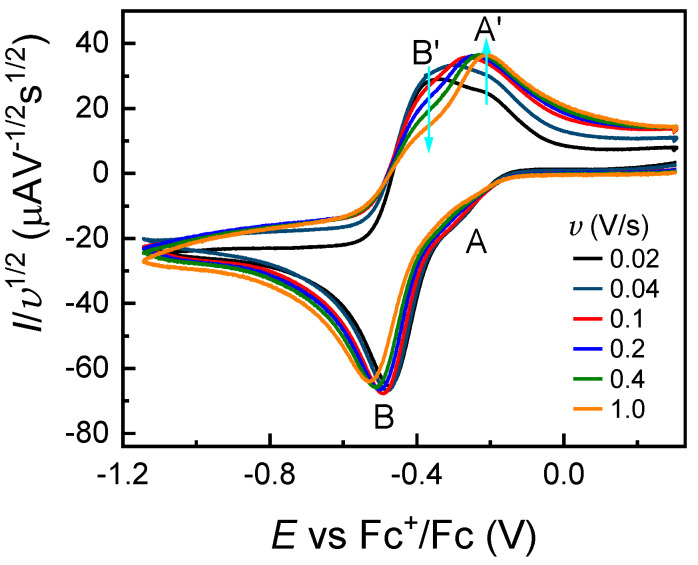
Cyclic voltammetry of 1 mM FeCl_3_ in DMF + 0.1 M Et_4_NBF_4_ recorded on a GC electrode at different scan rates at *T* = 25 °C.

Figure 1 shows cyclic voltammetry (CV) of FeCl_3_ in DMF + 0.1 M Et_4_NBF_4_, recorded at different scan rates. A well-defined cathodic peak (B) around −0.5 V and a shoulder (A) around −0.2 V are observed in the cathodic scan, while two anodic peaks, labelled A′ and B′, are observed upon scan reversal. The voltametric behavior of the iron salt strongly depends on scan rate (*v*). The cathodic peak B and the shoulder shift to more negative potentials when *v* is increased while the normalized peak current of peak B, *I*_p_(B)/*v*^1/2^, shows only a slight decrease. Conversely, major changes with *v* occur in the reverse scan. As *v* increases, peak A′ increases at the expense of peak B′, which instead decreases. It is clear that more than one Fe(III) species is present in solution and, above all, the electrogenerated Fe(II) is distributed in different species with distinct oxidation potentials.

**Figure 2:**
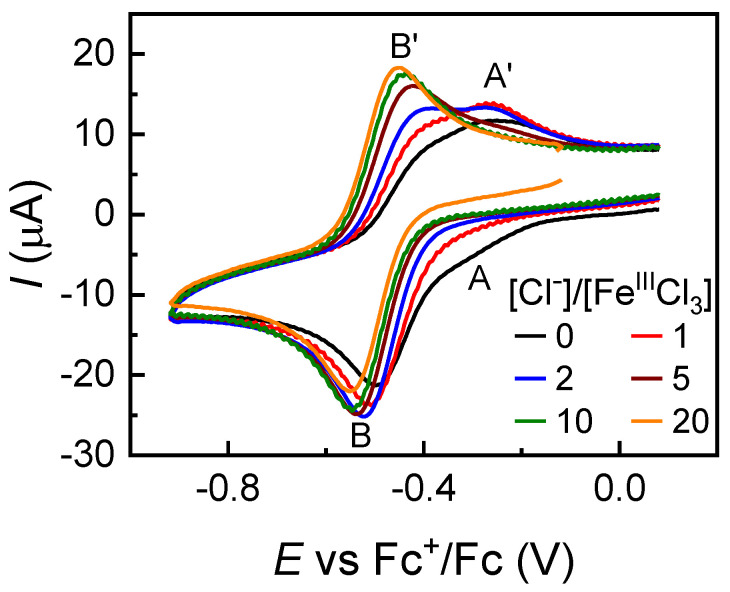
Cyclic voltammetry of 1 mM FeCl_3_ in DMF + 0.1 M Et_4_NBF_4_ recorded on a GC electrode at *v* = 0.2 V/s and *T* = 25 °C, before and after addition of different amounts of Et_4_NCl.

The dependence of the relative intensities of peaks A′ and B′ on scan rate points out the existence of chemical reactions between the Fe(II) species involved in the oxidation processes at these peaks. Before making any hypothesis on the identity of these Fe(II) species, we attempted to identify the principal Fe(III) species present in solution. To this end, CV of a DMF solution of FeCl_3_ was investigated in the presence of added chloride ions. As shown in Figure 2, both the shoulder (A) and the anodic peak A′ decreased and eventually disappeared as [Cl^−^] was increased. When a large excess of Cl^−^ was added, a single peak couple (B/B′) remained. This peak couple can be assigned to the FeCl_4_^−^/FeCl_4_^2−^ redox couple. The separation between the anodic and cathodic peak potentials, Δ*E*_p_ = *E*_p,B′_ – *E*_p,B_, is 71 mV at *v* = 0.01 V/s and increases with scan rate (at *v* = 1 V/s, Δ*E*_p_ = 118 mV). Therefore, the redox process under the peak couple is a quasi-reversible electron transfer. The half-wave potential calculated as the mid-point between the cathodic and anodic peaks, *E*_1/2_ = (*E*_p,B′_ + *E*_p,B_)/2 does not depend on *v* and the average value measured at different scan rates is −0.499 ± 0.003 V vs. Fc^+^/Fc.

When FeCl_3_ is dissolved in DMF, it undergoes speciation into FeCl_4_^−^, FeCl_2_^+^ and arguably some other Fe(III) species. Dass and George [56] investigated the speciation of iron(III)-chloro complexes in DMF by ultraviolet absorption spectroscopy. They concluded that dissolving FeCl_3_ in DMF produces mainly FeCl_4_^−^ and FeCl_2_^+^ together with small amounts of FeCl^2+^. Kim and Park [57] investigated the structure of the adduct obtained by complexation of FeCl_3_ with DMF. They prepared the adduct by dissolving ferric chloride in DMF, followed by slow vacuum evaporation of excess DMF. After careful analysis of the solid with various techniques, they assigned the chemical formula [Fe^III^Cl_2_(DMF)_1.2_(H_2_O)_2.7_]^+^[Fe^III^Cl_4_(DMF)_2.1_]^−^ to the adduct. Although the adduct was isolated only in the solid state, it likely arises from dichloro- and tetrachloro-iron(III) species in solution, which crystallize as an adduct when all excess solvent molecules are removed by evaporation. This study also confirmed that the preferred coordination number of iron(III) is 6. Throughout this paper, we will assume that all iron halide species are hexacoordinated, but for the sake of simplicity, we will omit coordinating solvent molecules.

**Figure 3:**
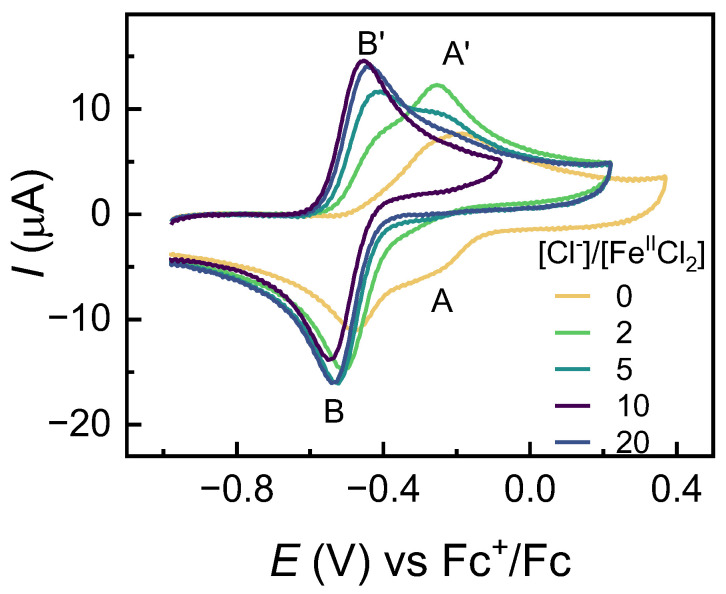
Cyclic voltammetry of 1 mM FeCl_2_ in DMF + 0.1 M Et_4_NBF_4_ recorded on a GC electrode at *v* = 0.2 V/s and *T* = 25 °C, before and after addition of different amounts of Et_4_NCl.

Interestingly, CV of FeCl_2_ showed almost the same pattern previously observed for FeCl_3_, except that the process now starts with the oxidation of Fe(II) to Fe(III) species. A broad anodic peak standing for two ill-resolved peaks is observed in the initial positive-going scan, while two well-defined peaks are observed in the reverse cathodic scan (Figure 3). When FeCl_2_ is dissolved in DMF, possibly the principal species present in solution is FeCl_2_ with minor formation of FeCl_4_^2−^ so that the main anodic peak is A′. After oxidation, the electrogenerated iron(III) species will undergo a new speciation equilibrium with significant presence of both FeCl_4_^−^ and FeCl_2_^+^. Reduction of this mixture in the reverse scan shows both peaks A and B. When increasing amounts of Cl^−^ were added, the B/B′ peak couple progressively increased while A/A′ decreased and disappeared in the presence of excess Cl^−^ (Figure 3). The peak couple obtained from FeCl_2_ with excess Cl^−^ had the same characteristics of that obtained from FeCl_3_ in the same conditions, indicating that the same redox couple is involved in both cases.

The set of analyses so far discussed allows for unambiguous assignment of peak couple B/B′ to FeCl_4_^−^/FeCl_4_^2−^. Considering the well-documented presence of FeCl_2_^+^ in DMF solutions of FeCl_3_ and the voltametric pattern of FeCl_2_, we assign peaks A/A′ to the FeCl_2_^−^/FeCl_2_ redox couple.

FeCl_2_^+^ + e^−^ ⇄ FeCl_2_ (peak couple A/A′) (1)

FeCl_4_^−^ + e^−^ ⇄ FeCl_4_^2−^ (peak couple B/B′) (2)

Let us now discuss the unusual scan-rate dependence of peaks A′ and B′ in Figure 1. The B/B′ peak couple is partially reversible at low scan rates, which can be taken to be indicative of partial disappearance of FeCl_4_^2−^ during the scan. One would then expect to see more reversibility when the scan rate is increased and, hence, the overall electrode reaction time is reduced. Surprisingly, the opposite scenario is observed: the peak couple tends toward full irreversibility at higher scan rates, while peak A′ increases (Figure 1). To rationalize this behavior, we have to consider a fast equilibrium between two iron(II) species:

FeCl_4_^2−^ ⇄ FeCl_2_ + 2Cl^−^ (3)

If this equilibrium is fast and well shifted to the right, FeCl_4_^2−^ will be converted to FeCl_2_ as soon as it is generated by electroreduction of FeCl_4_^−^ at peak B. At high scan rates, the voltametric pattern will be more akin to the equilibrium situation: small equilibrium FeCl_4_^2−^ concentration, small or hardly perceptible anodic peak B′. At low scan rates, the overall oxidation process at peak B′ continues occurring for a much longer time. When the small quantity of FeCl_4_^2−^ initially present at equilibrium is consumed, reaction (3) is shifted to the left to restore the equilibrium, but FeCl_4_^2−^ continues to be oxidized, producing, at the end, a signal much higher than predicted according to the equilibrium concentration of FeCl_4_^2−^. In other words, the anodic peak B′ arises from a sequence of two reactions, as shown in Equation (4). Indeed, this sequence is at play also at high scan rates, but its effect is negligible because the scan rate is so high that the experiment ends before FeCl_4_^2−^ is regenerated to any appreciable extent by reaction (3). Iron(II) is present in solution either as FeCl_4_^2−^ or FeCl_2_. At low scan rates, the kinetics of equilibrium (3) affects the overall oxidation process and peak B′ becomes more prominent than peak A′. Conversely, when at high scan rates, the effect of the backward reaction in Equation (3) becomes negligible, the oxidation process is more representative of the equilibrium conditions and A′ becomes the only observable anodic peak.

FeCl_2_ + 2Cl^−^ ⇄ FeCl_4_^2−^ ⇄ FeCl_4_^−^ + e^−^ (4)

Starting from either FeCl_3_ or FeCl_2_, it is possible to prepare DMF solutions containing exclusively FeCl_4_^−^ or FeCl_4_^2−^ and determine the redox properties of FeCl_4_^−^/FeCl_4_^2−^. Independent experiments on FeCl_3_ and FeCl_2_ performed in DMF + 0.1 M Et_4_NBF_4_ in the presence of a large excess of Cl^−^ gave *E*_1/2_ = −0.499 ± 0.003 V vs. Fc^+^/Fc and diffusion coefficients, *D*, of (8.0 ± 0.8) × 10^−6^ cm^2^ s^−1^ and (2.3 ± 0.1) × 10^−6^ cm^2^ s^−1^ for FeCl_4_^−^ and FeCl_4_^2−^, respectively (Figures S1 and S2). The diffusion coefficients were calculated from cyclic voltammetry according to the equation of Randles–Sevcik [58]. These data can be used to determine the standard potential of the redox couple according to Equation (5):

(5) $$E_{1/2}=E{{}^{\circ}}^{\prime }+\frac{RT}{F}ln{\left(\frac{D_{\mathrm{Red}}}{D_{\mathrm{Ox}}}\right)}^{1/2}$$

where *E*°′ is the formal potential, *R* is the universal gas constant, *F* is the Faraday constant and Ox and Red stand for the oxidized and reduced forms of the redox couple. Approximately assuming *E*° = *E*°′ gave *E*° = −0.483 ± 0.003 V vs. Fc^+^/Fc. The standard electron transfer rate constant, *k*°, was determined by the method of Nicholson [59]. Assuming a value of 0.5 for the transfer coefficient, α, Δ*E*_p_ values obtained from CVs of both FeCl_4_^−^ and FeCl_4_^2−^ were fit to the theoretical working curve of Nicholson (Figure S3). Both data series showed excellent fits and produced an average *k*° value of (1.21 ± 0.36) × 10^−2^ cm s^−1^.

### 2.2. Cyclic Voltammetry of Chloro(2-pyridylamino-N,N-bis(2-methylene-4,6-dichlorophenolate))iron(III), Fe^III^L(Cl)

**Figure 4:**
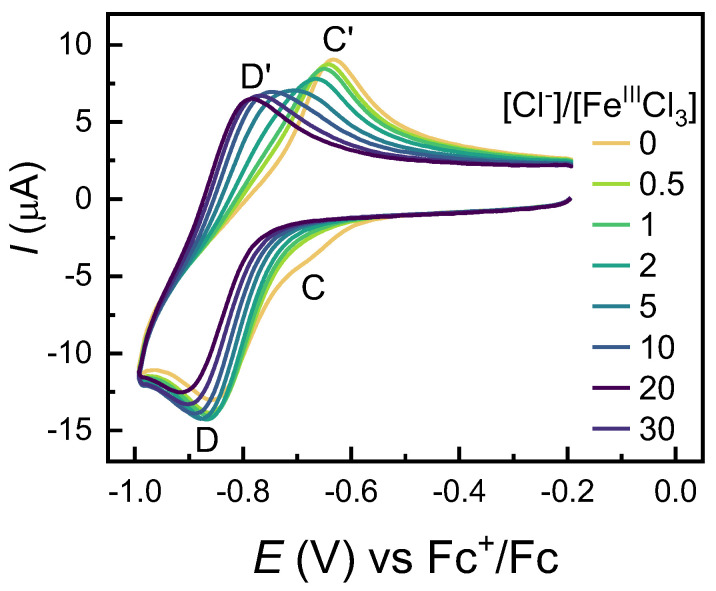
Cyclic voltammetry of 1 mM Fe^III^L(Cl) in DMF + 0.1 M Et_4_NBF_4_ recorded on a GC electrode at *v* = 0.2 V/s and *T* = 25 °C, before and after addition of different amounts of Et_4_NCl.

The amine–bis(phenolate) complex Fe^III^L(Cl) in solution likely exhibits a trigonal bipyramidal five-coordinate geometry, with four coordination sites occupied by L and one by the chlorine anion or by a solvent molecule [60]. Cyclic voltammetry of Fe^III^L(Cl) shows two cathodic peaks labeled C and D and only one anodic peak (labeled C′) in the reverse scan (Figure 4). This pattern did not change when the scan rate was increased. When instead excess Cl^−^ was added to the solution, peaks C and C′ disappeared while a new anodic peak (labeled D′) appeared. The voltametric behavior of Fe^III^L(Cl) can be rationalized by considering the presence of a small amount of Fe^III^L^+^ together with Fe^III^L(Cl), which is the principal Fe(III) species present in solution. When Cl^−^ is added in large excess over Fe(III), Fe^III^L^+^ is converted to Fe^III^L(Cl). Therefore, peaks C and D can be attributed to the reduction of Fe^III^L^+^ and Fe^III^L(Cl), respectively. The assignment of the anodic peaks D′ and C′ is also straightforward. They are coupled with the cathodic peaks C and D, respectively. Thus, the observed voltametric pattern stands for the following one-electron redox reactions:

Fe^III^L^+^ + e^−^ ⇄ Fe^II^L (peak couple C/C′) (6)

Fe^III^L(Cl) + e^−^ ⇄ Fe^II^L(Cl)^−^ (peak couple D/D′) (7)

Fe^III^L^+^ arises from partial dissociation of Fe^III^L(Cl) in DMF, but a small amount of Cl^−^ is enough to convert all Fe^III^L^+^ in solution into Fe^III^L(Cl). Indeed, peak C almost disappears as soon as the [Cl^−^] / [Fe^III^L(Cl)] reaches 0.5. Notably, however, the appearance of peak D′ and its development to a full peak, as well as the disappearance of peak C′, require addition of a large excess of Cl^−^ over Fe^III^L(Cl). Fe^II^L(Cl)^−^ generated in reaction (7) rapidly and reversibly dissociates to Fe^II^L and Cl^−^ (Equation (8)). Thus, when low [Cl^−^] / [Fe^III^L(Cl)] is used, the oxidation peak of Fe^II^L remains well evident, even if there is no Fe^III^L^+^ in solution (peak C is absent). A large excess of Cl^−^ is required to fully suppress the dissociation reaction and set the conditions for one-electron reduction of Fe^III^L(Cl) without complications due to a chemical reaction following the electron transfer.

Fe^II^L(Cl)^−^ ⇄ Fe^II^L + Cl^−^ (8)

The redox properties of Fe^III^L(Cl) were further investigated to determine *E*° and *k*° in DMF in the presence of Cl^−^ with [Cl^−^] / [Fe^III^L(Cl)] = 30. A quasi-reversible peak couple with scan rate-dependent Δ*E*_p_ was observed (Figure S4). The half-wave potential calculated as the average of the values measured at different scan rates is −0.853 ± 0.001 V vs. Fc^+^/Fc. Assuming similar diffusion coefficients for Fe^III^L(Cl) and Fe^II^L(Cl)^−^ and neglecting the activity coefficient contribution in Equation (5), the measured *E*_1/2_ can be approximately taken as *E*°. The standard heterogeneous rate constant of electron transfer was determined by the method of Nicholson [59], assuming *α* = 0.5. The best fit of the experimental data on the working curve (Figure S5) gave *k*° = (1.10 ± 0.04) × 10^−3^ cm s^−1^.

### 2.3. Cyclic Voltammetry of FeCl_3_ in the Presence of Other Ligands

Phosphorous- and nitrogen-based ligands are often used as additional ligands in FeX_3_-catalyzed ATRP. FeX_3_ is often mixed with a ligand L with the assumption that a more active catalyst species FeX_3_/L is formed. We investigated the role of triphenylphosphine (TPP) and tris(2-pyridylmethyl)amine (TPMA), taken as examples of phosphorous and nitrogen ligands, respectively, on the redox behavior of FeCl_3_.

**Figure 5:**
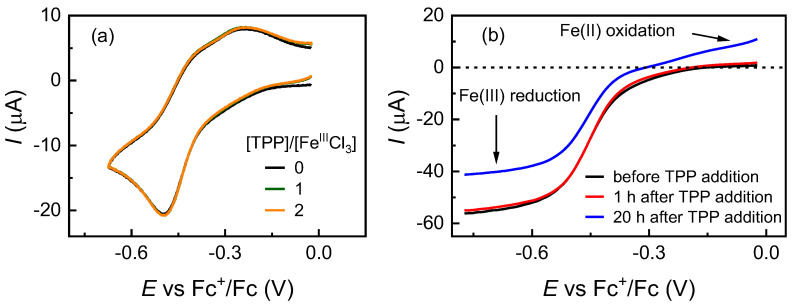
Voltammetry of 1.16 mM FeCl_3_ in DMF + 0.1 M Et_4_NBF_4_ recorded on a GC electrode at *T* = 25 °C; (**a**) CV recorded at *v* = 0.2 V/s before and after addition of TPP; (**b**) linear sweep voltammetry on a rotating disc electrode at *ω* = 2000 rpm and *v* = 0.005 V/s, recorded before and after addition of a 2-fold excess of TPP.

Figure 5a shows CVs recorded for FeCl_3_ before and after addition of TPP. No change in the voltametric pattern of the iron chloride complex(es) was observed. A similar result was obtained by UV-vis analysis. The absorption spectrum recorded after addition of TPP was simply the superimposition of the separate spectra of FeCl_3_ and TPP (Figure S6). The voltametric pattern of FeCl_2_ was also unaffected by TPP (Figure S7). These results clearly show that TPP does not form new complexes with FeCl_3_ or FeCl_2_ in the investigated reaction conditions. Walker and Poli [61] reported the synthesis of FeCl_3_-phosphine complexes of general formula FeCl_3_(PR_3_)_2_ (R = Me, Ph, cyclohexyl). The compounds were prepared at low temperature in nonpolar solvents, such as benzene and toluene, but their solutions were unstable at room temperature or higher. Phosphine complexes of FeX_2_ were reported by Sawamoto and co-workers as FeX_2_(L_2_) (X = Cl, L = PMePh_2_; X = Br, L = PMePh_2_, PPh_3_, P(*n*-Bu)_3_) [34]. They were prepared in toluene and used as ATRP catalysts in the same solvent at 80 °C. Although these complexes seem to be stable in these reaction conditions, their stability and chemical structure in polar solvents, such as DMF, are yet to be proved.

To check whether TPP can reduce Fe(III) to Fe(II), a mixture of FeCl_3_ with a two-fold excess of TPP in DMF + 0.1 M Et_4_NBF_4_ was monitored by linear sweep voltammetry (LSV) at a rotating disc electrode at room temperature. LSV recorded at *t* = 0 was identical with that observed before TPP addition and showed a well-defined wave for the reduction of Fe(III) to Fe(II) with a cathodic limiting current, |*I*_L,c_| = 56 μA. Since the system initially contained only Fe(III), the anodic limiting current, *I*_L,a_, was zero. The LSV recorded after 1 h showed a slight decrease in |*I*_L,c_| together with small *I*_L,a_. The reaction was left overnight and after 20 h, a significantly decreased Fe(III) concentration accompanied by the build-up of Fe(II) was observed, clearly indicating that FeCl_3_ was slowly reduced by TPP. The ability of triphenylphosphines to act as reducing agents in iron-catalyzed ATRP has previously been suggested and, in a few cases, experimentally demonstrated [62,63]. The reaction between FeCl_3_ and TPP is very slow at room temperature and probably will not affect the rate of polymerization in such conditions. However, many Fe-catalyzed polymerizations, especially in the case of MMA and styrene, are typically carried out at temperatures as high as 110 °C, where Fe(III) reduction by phosphines might be fast.

**Figure 6:**
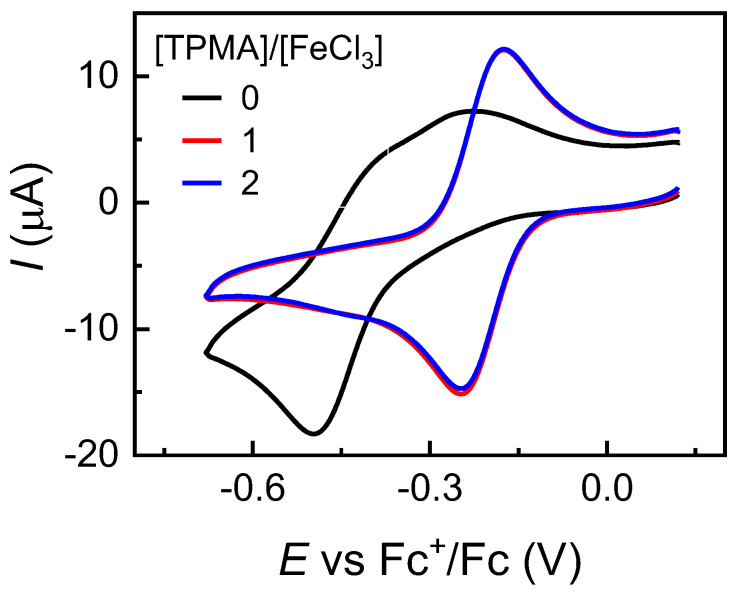
Cyclic voltammetry of 1.1 mM FeCl_3_ in DMF + 0.1 M Et_4_NBF_4_ recorded on a GC electrode at *v* = 0.2 V/s and *T* = 25 °C, before and after addition of TPMA.

In contrast to TPP, the nitrogen ligand TPMA reacted rapidly and quantitatively with FeCl_3_. Addition of 1 equiv. of TPMA to a solution of FeCl_3_ caused full disappearance of the original signal, which was replaced by a reversible peak couple at a less negative potential (Figure 6). Further addition of TPMA did not affect the voltametric response. It is obvious that a new complex, in which both Fe(III) and Fe(II) are stable, is formed. TPMA complexes of both Fe(III) and Fe(II) are known in the literature [64,65]. They have a distorted octahedral geometry arising from tetra-coordination by TPMA plus coordination of chloride ions, FeCl_2_(TPMA)^+^ and FeCl_2_(TPMA). Therefore, reactions (9) and (10) occur when TPMA is added to a DMF solution of FeCl_3_. The standard reduction potential of FeCl_2_(TPMA)^+^ (Equation (11)) can be estimated as *E*° ≈ *E*_1/2_ = (*E*_pa_ + *E*_pc_)/2, where *E*_pa_ and *E*_pc_ are the anodic and cathodic peak potentials and the measured value is *E*_1/2_ = −0.211 V vs. Fc^+^/Fc.

FeCl_4_^−^ + TPMA ⇄ FeCl_2_(TPMA)^+^ + 2Cl^−^ (9)

FeCl_2_^+^ + TPMA ⇄ FeCl_2_(TPMA)^+^ (10)

FeCl_2_(TPMA)^+^ + e^−^ ⇄ FeCl_2_(TPMA) (11)

We examined the effect of Cl^−^ excess on the stability of FeCl_2_(TPMA)^+^. Addition of a two-fold excess of Cl^−^ over FeCl_3_ did not affect the CV pattern of the iron–TPMA complex (Figure S8). A decrease in the cathodic peak of FeCl_2_(TPMA)^+^ together with the appearance of the cathodic peak of FeCl_4_^−^ was observed only when a large excess of Cl^−^ was added. This indicates that Fe(III) has a much higher affinity for TPMA than Cl^−^, but reaction (9) shifts to the left as the concentration of added Cl^−^ is increased. Notably, when in the presence of excess Cl^−^, the principal iron species present in solution became FeCl_4_^−^, a reversible peak couple for the FeCl_4_^−^ / FeCl_4_^2−^ couple was not observed (Figure S8). This means that the stability constant of FeCl_2_(TPMA) is higher than that of FeCl_2_(TPMA)^+^. Once FeCl_4_^−^ is reduced to FeCl_4_^2−^, the latter rapidly reacts with TPMA to form FeCl_2_(TPMA), so that the oxidation peak of FeCl_4_^2−^ is missing while the anodic peak due to FeCl_2_(TPMA) oxidation is always present (Figure S8).

In summary, the Fe(III) species formed in solution and their standard reduction potentials are summarized in Table 1. When no ligand is added or TPP is used as a ligand, the principal Fe(III) complex in solution is FeCl_4_^−^ with a characteristic *E*° of −0.483 V vs. Fc^+^/Fc. 2-pyridylamino-*N*,*N*-bis(2-methylene-4,6-dichlorophenolate) (L) forms a stable complex, Fe^III^L(Cl), with a cathodic shift of *E*° of Fe(III)/Fe(II) by 0.37 V. TPMA also forms a stable complex with the iron salt, but the standard potential of the Fe(III)/Fe(II) couple shifts anodically by 0.272 V. In ATRP, a metal at a low oxidation state (Fe(II) in this case) reacts with an alkyl halide initiator, RX or a halogen-capped dormant species, P_n_-X, to generate the active radical species. The rate of this activation reaction is strongly dependent on *E*° of the metal complex. Therefore, the expected order of activity of the Fe complexes examined here is Fe^III^L(Cl) > Fe^III^Cl_4_^−^ > Fe^III^Cl_2_(TPMA)^+^. However, in addition to the ability to activate RX, an efficient ATRP catalyst must also be a good radical deactivator; hence, predicting the overall efficacy of an iron catalyst, on the basis of *E*° alone, is not straightforward.

### 2.4. Electrochemically Mediated ATRP (eATRP)

#### 2.4.1. eATRP Mediated by Amine–bis(phenolate) iron(III) Chloride, Fe^III^L(Cl)

The voltametric behavior of Fe^III^L(Cl) in 50 vol% MMA in DMF + 0.1 M Et_4_NBF_4_ is slightly different from that in pure DMF. The pre-peak C is absent, whereas peak D shows partial reversibility (Figure S9). It appears that dissociation of Fe^III^L(Cl) to give Fe^III^L^+^ and Cl^−^ is disfavored in the monomer/solvent mixture. Further, Fe^II^L(Cl)^−^ is more stable in MMA/DMF than in pure DMF. These changes are likely due to modifications of medium polarity, which decreases when MMA with a dielectric constant, ε, of 6.53 at 25 °C [66] is added to DMF (ε = 38.25 at 20 °C [67]). The mixture has lower ability to solvate ions than pure DMF and, therefore, dissociation of Fe^III^L(Cl) and Fe^II^L(Cl)^−^ becomes less favored in the mixture.

*e*ATRP of MMA mediated by Fe^III^L(Cl) was carried out in 50/50 (*V/V*) DMF/MMA mixtures with 5 mM catalyst and 15 mM initiator. As initiators, ethyl α-chlorophenyl acetate, ECPA, and ethyl α-bromophenyl acetate, EBPA, which are among the most active alkyl halides used in ATRP, were chosen. Nonetheless, CV tests revealed that electrogenerated Fe^II^L(Cl)^−^ reacts very slowly with these initiators (Figure S9). Since it was not easy to estimate *E*_1/2_ of the catalyst in the typical reaction conditions, the applied potential, *E*_app_, was chosen with reference to the cathodic peak potential of the mediator (peak D). The reaction time was set at 4 h, but in some cases, polymerization was stopped earlier (2–3 h) because of drastic increase in viscosity.

**Figure 7:**
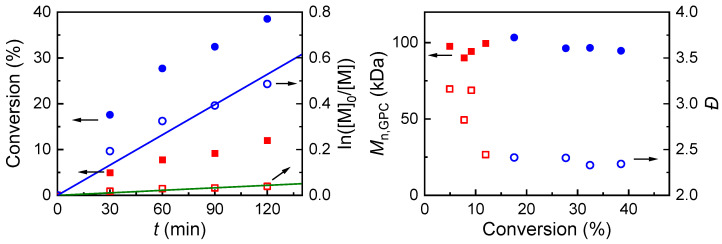
*e*ATRP of MMA mediated by Fe^III^L(Cl) in DMF/MMA (50/50, *V*/*V*) + 0.1 M Et_4_NBF_4_ at *T* = 50 °C, with ECPA as initiator (squares) at *E*_app_ = *E*_p_ or EBPA as initiator (circles) at *E*_app_ = *E*_p_ + 0.06 V. Other conditions: [MMA]:[RX]:[Fe^III^L(Cl)] = 100:0.32:0.11 with [Fe^III^L(Cl)] = 5 mM.

The results of the electrochemically mediated polymerizations are collected in Table 2, whereas examples of reaction kinetics and trends of *M*_n_ and dispersity are shown in Figure 7. Using ECPA at 70 °C and *E*_app_ = *E*_p_, 42.3% conversion was achieved after 4 h, but the reaction was not controlled. The dispersity of the polymer was very high and its molecular weight exceeded the theoretical value by about one order of magnitude (Table 2, entry 1). This first experiment indicated that activation was effective, but deactivation was inefficient. The experiment was then repeated with a 10-fold excess of Cl^−^ with respect to the iron mediator to increase the concentration of the deactivator Fe^III^L(Cl). Again, the reaction was fast without any control (entry 2). Last, the temperature was lowered to 50 °C. The reaction was slower, but the dispersity remained very high and *M*_n_ was 20-fold higher than the theoretical value (entry 3). Two more experiments were performed with this mediator by changing the initiator to EBPA. Activation of this initiator by ATRP mechanism will produce Fe^III^L(Br), which might be a better deactivator than Fe^III^L(Cl). Disappointingly, the results were very similar to those obtained with ECPA (Table 2, entries 3–4 and Figure 7).

Overall, the amine–bis(phenolate) iron(III) complex proved to be inefficient as a mediator of controlled radical polymerization of MMA in DMF under electrochemical generation of Fe(II) activator species. This result is at variance with previous reports by Shaver and co-workers on well-controlled polymerizations of MMA and styrene mediated by various amine–bis(phenolate) iron(III) complexes in the presence of radical initiators, such as azobis(isobutyronitrile) [37,68]. There are, however, important differences between the experimental conditions. All previous polymerizations were performed in bulk or in mixtures of monomer with nonpolar solvents. Further, the typical concentration of the iron(III) complex was nearly 100 mM. Last, the reactions were carried out at temperatures as high as 120 °C. Obviously these conditions are not desirable, especially the use of high catalyst concentration, which inevitably will involve costly polymer purification steps. *e*ATRP with this type of mediator was here investigated to explore the possibility of employing a low-catalyst load under mild conditions. The failure of control in low-catalyst-load *e*ATRP can be attributed to lack of deactivation. Schroeder and Buback [69] measured deactivation rate constants, *k*_deact_, in iron-mediated ATRP. For an amine–bis(phenolate) iron(III) complex, they measured at 60 °C a *k*_deact_ value of about 10^4^ M^−1^ s^−1^, which is 2 orders of magnitude lower than *k*_deact_ of copper complexes. This value increases by one order of magnitude if the temperature is raised to 120 °C. While deactivation rate in bulk monomer at 120 °C with high catalyst load is high enough to ensure control over molecular weight distribution, it is inefficient in *e*ATRP. Indeed, due to lower *T* and lower catalyst loading, it can be estimated that deactivation rate in the *e*ATRP experiments is at least 2 orders of magnitude lower than in the reaction conditions employed by Shaver and co-workers.

#### 2.4.2. eATRP Mediated by FeCl_3_

*e*ATRP of MMA mediated by iron halide complexes, both in the absence and presence of a phosphorous ligand, has already been reported [44,45,46]. These studies used FeBr_3_ and FeCl_3_.6H_2_O with tris(2,4,6-methoxyphenyl)phosphine as an additional ligand or FeCl_3_.6H_2_O without added ligand and, in most cases, the best results were obtained when quite high concentrations of iron salt were used. For comparison with FeBr_3_ and FeCl_3_.6H_2_O, which was the principal catalyst in their study, Wang et al. [46] reported a single *e*ATRP experiment with FeCl_3_ [46]. Using ca 31 mM FeCl_3_ in *N*-methylpyrrolidone at 95 °C, they achieved 43% monomer conversion after ca 6.5 h, obtaining a polymer with dispersity *Ð* = 1.34.

**Figure 8:**
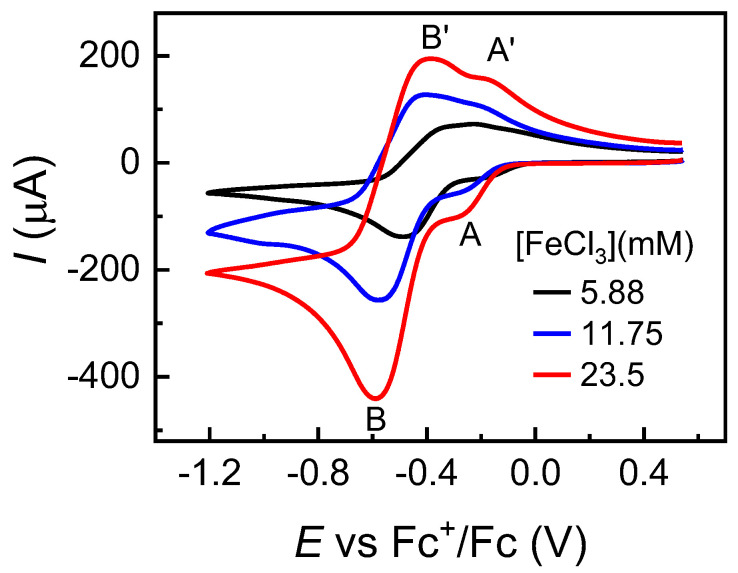
Cyclic voltammetry of FeCl_3_ in DMF/MMA (50/50, *V*/*V*) + 0.1 M Et_4_NBF_4_ recorded on a GC electrode at *v* = 0.2 V/s and *T* = 55 °C.

Based on these previous studies, a relatively high concentration of FeCl_3_ was first considered. Before *e*ATRP experiments, the effect of [FeCl_3_] and monomer on the voltametric behavior of Fe(III) was examined in conditions of *e*ATRP. Addition of MMA to DMF did not change the general CV pattern of FeCl_3_, but peak A′ became more pronounced while peak B gained more reversibility. The equilibrium distributions of both Fe(III) and Fe(II) species are influenced by medium polarity as well as initial FeCl_3_ concentration and temperature. The effect of [FeCl_3_] is evidenced in Figure 8, which shows a remarkable enhancement of peak B′ as [FeCl_3_] is increased. In DMF/MMA, it is possible to estimate *E*_1/2_ of the FeCl_4_^−^ / FeCl_4_^2−^ redox couple as (*E*_p,B_ + *E*_p,B′_)/2, especially at high [FeCl_3_] and low scan rates. The estimated value was −0.49 V vs. Fc^+^/Fc.

**Figure 9:**
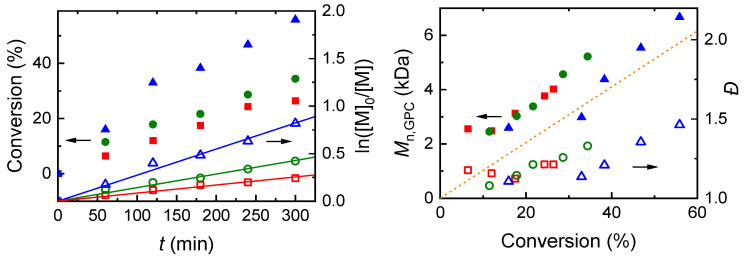
*e*ATRP of MMA mediated by FeCl_3_ in DMF/MMA (50/50, *V/V*) + 0.1 M Et_4_NBF_4_ at 70 °C and *E*_app_ = *E*_1/2_ (squares), *E*_app_ = *E*_p_ − 0.06 V (circles), and *E*_app_ = *E*_p_ − 0.12 V (triangles). The dashed line stands for theoretical *M*_n_. Other conditions: [MMA]:[EBPA]:[FeCl_3_] = 100:1:0.5 with [FeCl_3_] = 23.5 mM.

The results of *e*ATRPs for MMA performed at different conditions are summarized in Table 3. The applied potential, *E*_app_, is reported as *E*_app_ − *E*_1/2_, with reference to the value of *E*_1/2_ measured from CV before electrolysis. A first set of *e*ATRPs at 70 °C was carried out with 23.5 mM FeCl_3_ at different *E*_app_ values. All polymerizations were well controlled, obeying first-order kinetic rate laws and producing polymers with narrow molecular weight distribution, though the experimental molecular weights did not perfectly match the theoretical values (Figure 9). *M*_n,GPC_ was always greater than *M*_n,th_, indicating initiation efficiency < 1. The rate of polymerization was higher at lower *E*_app_ values, but polymer dispersity was better at higher (less negative) applied potentials (Table 3, entries 1–3). Lowering the temperature to 55 °C at *E*_app_ = *E*_1/2_ − 0.06 V had no significant effect on reaction rate and polymer properties (Table 3, entry 4). Therefore, most of the other experiments were conducted at 55 °C.

Next, the effect of [FeCl_3_] and [EBPA]/[FeCl_3_] ratio was tested at *E*_app_ = *E*_1/2_ − 0.06 V. Lowering [FeCl_3_] from 23.5 mM to 11.75 mM without changing [EBPA] did not appreciably affect polymerization performance; only a slight increase in dispersity was observed (Table 3, entry 5). However, when [EBPA] was also halved, the reaction became slower and polymer dispersity increased to 1.55 (Table 3, entry 6). A further decrease in [FeCl_3_] to 5.88 mM with [EBPA] = 23.5 mM slightly decreased the dispersity (Table 3, entry 7). Interestingly, the reaction rate was similar with that of the experiment with [FeCl_3_] = 11.75 mM and [EBPA] = 47.5 mM (Table 3, entries 5 and 7).

In an attempt to improve polymerization control, the *e*ATRP experiment of entry 7 in Table 3 was repeated at *E*_app_ = *E*_1/2_. The dispersity was little affected (slight increase from 1.50 to 1.53) but the reaction rate decreased considerably, with conversion after 5 h dropping from 38.5% to 23.7%. The temperature was then raised to 70 °C to increase the reaction rate. As desired, the conversion increased more than twice while the dispersity remained unchanged (Table 3, entry 9).

Two more experiments were performed to test the role of added ligands. The *e*ATRP of entry 7 (Table 3) was repeated with a two-fold excess of TPP over FeCl_3_ (Table 3, entry 10). Conversion after 5 h increased from 38.5% to 40.6%, while the dispersity remained unchanged. We previously showed that TPP acts as a weak reducing agent rather than a ligand able to form a new complex with FeCl_3_ in DMF. The slight rate enhancement may be attributed to the contribution Fe(II) regeneration via reduction by TPP in the homogeneous phase. The same experiment was again repeated in the presence of TPMA in place of TPP (Table 3, entry 11). No polymerization was observed after 5 h, clearly indicating that FeCl_2_(TPMA) is not able to activate the initiator.

To sum up, ligand-free FeCl_3_ is a cheap ATRP catalyst able to achieve good polymerization control under appropriate conditions. The efficacy of the catalyst depends on many parameters, including concentrations of catalyst and initiator, type of solvent, temperature and applied potential. Further work systematically addressing the effects of these parameters and their combinations is necessary to define optimal controlled-polymerization conditions.

## 3. Materials and Methods

### 3.1. Materials

Anhydrous dimethylformamide (DMF) used as solvent in electrochemical investigations was purchased from Sigma-Aldrich, Darmstadt, Germany, (99.8%, anhydrous) and used without further purification. FeCl_3_ (Alfa Aesar, Kandel, Germany, 98%), FeCl_2_ (Alfa Aesar, 98%), tris(2-pyridylmethyl)amine (TPMA, Sigma-Aldrich, 98%), CDCl_3_ (Sigma-Aldrich, 99.8%, anhydrous), LiBr (Alfa Aesar, 99%), basic aluminum oxide (Al_2_O_3_, Honeywell Riedel-de Haën), DMF (Carlo Erba, Cornaredo, Italy 99.9%, HPLC), ethanol (Carlo Erba, 99.8%), ethyl ether (Sigma-Aldrich, 98%), ethyl α-bromophenylacetate (EBPA, Alfa Aesar, 97%) and H_2_SO_4_ (Fluka, Buchs, Switzerland, 95%, TraceSELECT) were used as received. Tetraethylammonium tetrafluoroborate (Et_4_NBF_4_, Alfa Aesar, 99%), used as a background electrolyte, was recrystallized twice from ethanol. Tetraethylammonium chloride (Sigma-Aldrich, 98%), used as a source of Cl^−^ ions in solution, was recrystallized from ethanol/ethyl ether. After recrystallization, both salts were dried in a vacuum oven at 70 °C for 48 h. Methyl methacrylate (Sigma-Aldrich, >99%) was percolated through a column of active basic aluminum oxide to remove polymerization inhibitors. 2-pyridylamino-*N*,*N*-bis(2-methylene-4,6-dichlorophenol) (H_2_L) and the iron(III) complex Fe^III^L(Cl) were prepared following a literature procedure [60] and their purity was checked by NMR (for H_2_L) and elemental analysis (Figure S12, Tables S1 and S2).

### 3.2. Instrumentation

Electrochemical measurements were carried out in a 5-neck glass cell under an Ar atmosphere; an Autolab PGSTAT 30 or 30N potentiostat/galvanostat (EcoChemie, Utrecht, The Netherlands) run by a PC with GPES or NOVA software (EcoChemie) was used. The working electrode, counter electrode and reference electrode used during voltametric investigations were a 3 mm diameter GC disk (Tokai GC-20), a Pt ring and Ag/AgI/0.1 M *n*-Bu_4_NI in DMF, respectively. Before each experiment, the GC disk was cleaned by polishing with a 0.25 µm diamond paste, followed by ultrasonic rinsing in ethanol for 5 min. The reference electrode was always calibrated with ferrocene (Fc), which was added at the end of each experiment as an internal standard and all potentials are reported versus the ferrocenium/ferrocene (Fc^+^/Fc) redox couple. Conversion of these potentials to the saturated calomel electrode scale can be achieved by using *E*^o^(Fc^+^/Fc) = 0.476 V vs. SCE [70].

*e*ATRP experiments were carried out in a two-compartment cell equipped with a Pt mesh (Alfa Aesar, 99.9% metals basis) working electrode, a graphite rod counter electrode and the same reference electrode used in cyclic voltammetry. Before each experiment, the Pt mesh was electrochemically activated in 0.5 M H_2_SO_4_ by cycling the potential from −0.7 V to 1 V vs. Hg/Hg_2_SO_4_ at a scan rate of 0.2 V s^−1^ (60 cycles). The counter electrode was separated from the working solution by a glass frit filled with the same electrolyte solution used in the working electrode compartment and a methylcellulose gel saturated with Et_4_NBF_4_.

Gel permeation chromatography (GPC) was used to determine the number average molecular weight (*M*_n_) and dispersity (*Đ*) of polymers prepared by *e*ATRP. The GPC instrument was Agilent 1260 Infinity, equipped with a refractive index (RI) detector and two PLgel Mixed-D columns (300 mm, 5 µm) connected in series. The column compartment and RI detector were thermostated at 70 °C and 50 °C, respectively. The eluent was DMF containing 10 mM LiBr, at a flow rate of 1 mL/min. Before injection, the samples were filtered through alumina over a PTFE membrane of 200 nm pore to remove any particulate material and the iron catalyst. The column system was calibrated with 12 linear poly(methyl methacrylate) standards (*M*_n_ = 540–2,210,000 Da). Monomer conversion was determined by ^1^H-NMR spectroscopy with a 200 MHz Bruker Avance instrument, using CDCl_3_ as a solvent.

UV-Vis spectra were recorded with an Agilent Cary 5000 spectrophotometer by using 10 mm optical path length quartz cuvettes.

### 3.3. eATRP of Methyl Methacrylate

A thermostated 5-neck electrochemical cell, flushed with an inert gas, was loaded with DMF/MMA (50:50, *V/V*) + 0.1 M Et_4_NBF_4_ and the desired amount of iron catalyst. After recording a CV of the catalyst, the initiator RX was injected, and a CV was recorded. Polymerization was then started by applying the selected applied potential (*E*_app_) and samples were withdrawn periodically to measure monomer conversion and *M*_n_ and *Đ* of the polymer.

## 4. Conclusions

In summary, we have shown that FeCl_3_ dissolves in DMF to yield FeCl_4_^−^ and FeCl_2_^+^. Reduction of these species gives prevalently FeCl_2_. A large excess of Cl^−^ is required to convert FeCl_3_ and FeCl_2_ to FeCl_4_^−^ and FeCl_4_^2^^−^, respectively. TPMA forms stable complexes with both Fe(III) and Fe(II). 2-Pyridylamino-*N*,*N*-bis(2-methylene-4,6-dichlorophenolate) (L) also gives a stable iron(III) complex, Fe^III^L(Cl), which provides Fe^II^L(Cl)^−^, upon one-electron reduction. In contrast, triphenylphosphine does not form a stable complex with FeCl_3_. It acts as a reducing agent. *e*ATRP of MMA catalyzed by Fe^III^L(Cl) was fast and uncontrolled. FeCl_3_ proved to be a much better catalyst than the amine–bis(phenolate) complex. FeCl_3_-mediated polymerization was well controlled, provided that a large amount of the iron salt was employed. No polymerization was observed when TPMA was used as an additional ligand. It appears that the best catalyst system is the ligand-free iron salt. A weakness of this system is in the deactivation step, characterized by a low rate constant.

## Figures and Tables

**Table 1:** Standard reduction potentials of Fe(III) complexes in DMF + 0.1 M Et_4_NBF_4_ at *T* = 25 °C.

Ligand	Complex	*E*° vs. Fc^+^/Fc (V)
-	FeCl_4_^−^	−0.483
L ^1^	FeL(Cl)	−0.853
TPP	FeCl_4_^−^	−0.483
TPMA	FeCl_2_(TPMA)^+^	−0.211

^1^ L = 2-pyridylamino-*N*,*N*-bis(2-methylene-4,6-dichlorophenolate).

**Table 2:** *e*ATRP of methyl methacrylate (MMA) in DMF/monomer (50/50, *V/V*) + 0.1 M Et_4_NBF_4_^1^.

Entry	Initiator	*E*_app_ − *E*_pc_ (V)	*T* (°C)	*t* (h)	*Q*/*Q*_th_ ^2^	Conv. ^3^ (%)	10^3^ *k*_app_^4^ (min^−1^)	*M*_n,GPC_^5^ (kDa)	*M*_n,th_^6^ (kDa)	*Đ* ^5^
1	ECPA	0	70	4	0.81	42.3	2.35	147.80	13.26	2.75
2 ^7^	ECPA	0	70	3	0.75	55.5	3.27	157.06	17.34	2.18
3	ECPA	0	50	4	0.70	17.6	0.36	109.04	5.64	2.54
4	EBPA	0.06	50	2	0.93	38.5	4.40	94.67	12.38	2.34
5	EBPA	−0.06	70	2	0.59	40.0	4.63	85.52	13.04	2.52

^1^ [MMA]:[RX]:[Fe^III^L(Cl)] = 100:0.32:0.11; [Fe^III^L(Cl)] = 5 mM, WE = Pt mesh, CE = graphite rod, *V* = 10 mL. ^2^ Ratio between passed charge and theoretical value calculated for 1e^-^ reduction of Fe(III). ^3^ Monomer conversion. ^4^ Apparent polymerization rate constant determined as the slope of ln([M]_0_/[M]) vs. *t*. ^5^ Determined by GPC. ^6^ Theoretical molecular weight. ^7^ In the presence of 0.05 M Et_4_NCl.

**Table 3:** *e*ATRP of methyl methacrylate (MMA) and butyl acrylate in DMF/monomer (50/50, *V*/*V*) + 0.1 M Et_4_NBF_4_ for 5 h ^1^.

Entry	[EBPA] (mM)	[FeCl_3_] (mM)	[M]:[I]:[Fe] ^2^	*E*_app_ − *E*_1/2_ (V)	*T* (°C)	*Q*/*Q*th ^3^ (C)	Conv. ^4^ (%)	10^3^ *k*_app_^5^ (min^−1^)	*M*_n,GPC_^6^ (kDa)	*M*_n,th_^7^ (kDa)	*Đ* ^6^
1	47.0	23.5	100:1:0.5	−0.12	70	1.08	55.9	27.4	6.67	5.84	1.46
2	47.0	23.5	100:1:0.5	−0.06	70	0.85	34.4	14.3	5.22	3.69	1.33
3	47.0	23.5	100:1:0.5	0.0	70	0.82	26.5	8.5	4.02	2.90	1.21
4	47.0	23.5	100:1:0.5	−0.06	55	0.93	38.3	16.8	6.37	4.33	1.36
5	47.0	11.75	100:1:0.25	−0.06	55	1.31	43.4	19.7	6.47	4.59	1.40
6	23.5	11.75	100:0.5:0.25	−0.06	55	1.23	32.6	13.5	10.24	6.77	1.55
7	23.5	5.88	100:0.5:0.126	−0.06	55	1.96	38.5	17.8	13.34	7.82	1.50
8	23.5	5.88	100:0.5:0.126	0.0	55	1.44	23.7	9.92	9.13	5.0	1.53
9	23.5	5.88	100:0.5:0.126	0.0	70	1.66	48.3	22.7	11.66	9.92	1.53
10 ^8^	23.5	5.88	100:0.5:0.126	−0.06	55	1.66	40.6	18.2	14.75	8.22	1.50
11 ^9^	23.5	5.88	100:0.5:0.126	−0.06	55	0.50	0	-	-	-	-

^1^ Other conditions: WE = Pt mesh, CE = graphite rod, *V* = 30 mL; [MMA] = 4.674 M. ^2^ [monomer]:[initiator]:[catalyst]. ^3^ Ratio between passed charge and theoretical value calculated for 1e^-^ reduction of Fe(III). ^4^ Monomer conversion. ^5^ Apparent polymerization rate constant determined as the slope of ln([M]_0_/[M]) vs. *t*. ^6^ Determined by GPC. ^7^ Theoretical molecular weight. ^8^ In the presence of a 2-fold excess of TPP with respect to FeCl_3_. ^9^ In the presence of a 2-fold excess of TPMA with respect to FeCl_3_.

## Data Availability

Data obtained in this study are available in the article and Supporting Materials.

## Supplementary Materials

The following supporting information can be downloaded online: https://www.mdpi.com/article/10.3390/molecules27196312/s1, Figure S1: CV of 0.93 mM FeCl_3_ in DMF + 0.1 M Et_4_NBF_4_ + 20 mM Et_4_NCl, recorded on a GC electrode at different scan rates at *T* = 25 °C; Figure S2: CV of 1.83 mM FeCl_2_ in DMF + 0.1 M Et_4_NBF_4_ + 20 mM Et_4_NCl, recorded on a GC electrode at different scan rates at *T* = 25 °C; Figure S3: fitting of experimental data obtained from CV of FeCl_4_^−^ and FeCl_4_^2−^ on a theoretical working curve (solid line) for the determination of *k*° in DMF + 0.1 M Et_4_NBF_4_ at *T* = 25 °C; Figure S4: CV of 1.0 mM Fe^III^L(Cl) in DMF + 0.1 M Et_4_NBF_4_ + 30 mM Et_4_NCl, recorded on a GC electrode at different scan rates at *T* = 25 °C; Figure S5: fitting of experimental data obtained from CV of Fe^III^L(Cl) on a theoretical working curve (solid line) for the determination of *k*° in DMF + 0.1 M Et_4_NBF_4_ at *T* = 25 °C; Figure S6: UV-vis spectra of 0.1 mM FeCl_3_, 0.2 mM TPP and a mixture of 0.1 mM FeCl_3_ + 0.2 mM TPP, in DMF; Figure S7: CV of 1.08 mM FeCl_2_ in DMF + 0.1 M Et_4_NBF_4_, recorded on a GC electrode in the absence and presence of triphenylphosphine (TPP) at *v* = 0.2 V/s and *T* = 25 °C; Figure S8: CV of 1.1 mM FeCl_3_ in DMF + 0.1 M Et_4_NBF_4_ recorded on a GC electrode at *v* = 0.2 V/s and *T* = 25 °C, before and after addition of TPMA and Et_4_NCl; Figure S9: CV of 5 mM Fe^III^L(Cl) in DMF/MMA (50/50, *V/V*) + 0.1 M Et_4_NBF_4_ recorded on a GC electrode at *v* = 0.2 V/s and *T* = 50 °C, in the absence (black line) and presence of 15 mM ECPA (blue line) or 15 mM EBPA (red line); Figure S10: GPC traces of PMMA samples taken at different monomer conversions during *e*ATRP of MMA mediated by Fe^III^L(Cl) in DMF/MMA (50/50, *V/V*) + 0.1 M Et_4_NBF_4_ with EBPA as initiator performed at 50 °C and *E*_app_ = *E*_p_ + 0.06 V; Figure S11: CV of 11.75 mM FeCl_3_ in DMF/MMA (50/50, *V/V*) + 0.1 M Et_4_NBF_4_ recorded on a GC electrode at *v* = 0.2 V/s and *T* = 55 °C, in the absence (black line) and presence of 47 mM EBPA (red line); Figure S12: ^1^H NMR spectrum (300 MHz, CDCl_3_) of 2-pyridylamino-*N*,*N*-bis(2-methylene-4,6-dichlorophenol); Table S1: ^1^H NMR spectral data of 2-pyridylamino-*N*,*N*-bis(2-methylene-4,6-dichlorophenol; Table S2: Elemental analysis of chloro(2-pyridylamino-*N*,*N*-bis(2-methylene-4,6-dichlorophenolate))iron(III). Ref. [60] is cited in Supplementary Materials.

## Sample Availability

Samples of the compounds are not available from the authors.
