# Supplementary material for: Electrochemical Investigation of Iron-Catalyzed Atom Transfer Radical Polymerization
Source: Molecules. 2022 Sep 24;27(19):6312. doi: 10.3390/molecules27196312 (PMC9570559; doi:10.3390/molecules27196312)
Supplement: Supplementary file 1 [file molecules-27-06312-s001.zip › molecules-1911755-supplementary.pdf]

Supporting Materials for

Electrochemical investigation of iron-catalyzed atom transfer radical polymerization

Gianluca Gazzola [1,†], Sebastiano Pasinato [1,†], Marco Fantin [1], Niccolò Braidi [2], Cristina Tubaro [1], Christian Durante [1] and Abdirisak Ahmed Isse [1,*]

[1] Department of Chemical Sciences, University of Padova, Via Marzolo 1, 35131 Padova, Italy
[2] Department of Chemical and Geological Sciences, University of Modena and Reggio Emilia, Via Campi 103, 41125 Modena, Italy
* Correspondence: abdirisak.ahmedisse@unipd.it
† These authors contributed equally to this work.

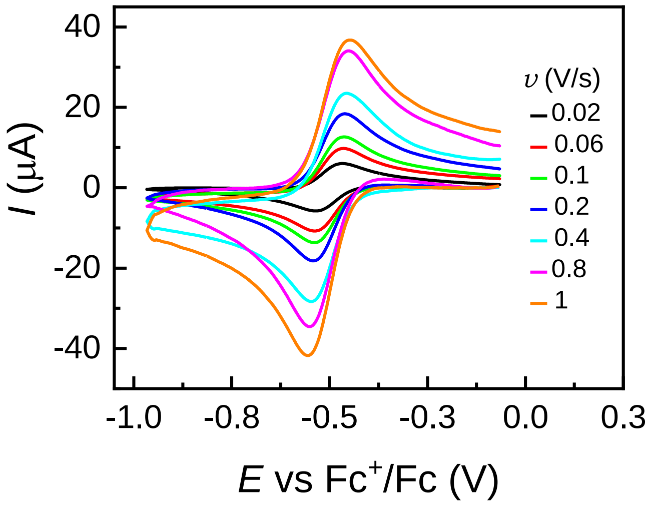

Figure S1. Cyclic voltammetry of 0.93 mM $FeCl_3$ in DMF + 0.1 M Et_4NBF_4 + 20 mM Et_4NCl, recorded on a GC electrode at different scan rates at T = 25 °C.

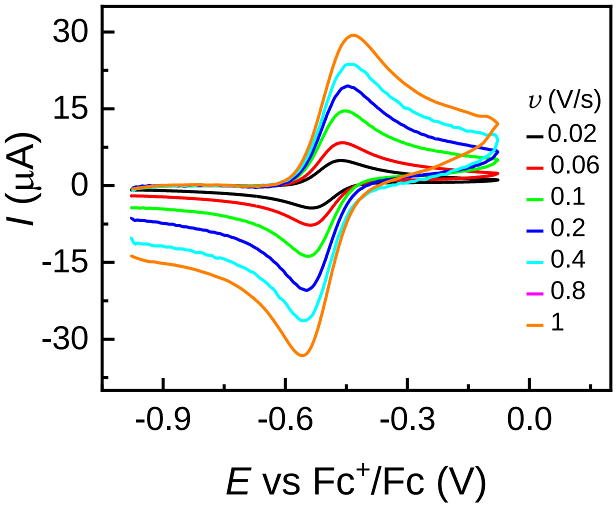

Figure S2. Cyclic voltammetry of 1.83 mM $FeCl_2$ in DMF + 0.1 M Et_4NBF_4 + 20 mM Et_4NCl, recorded on a GC electrode at different scan rates at T = 25 °C.

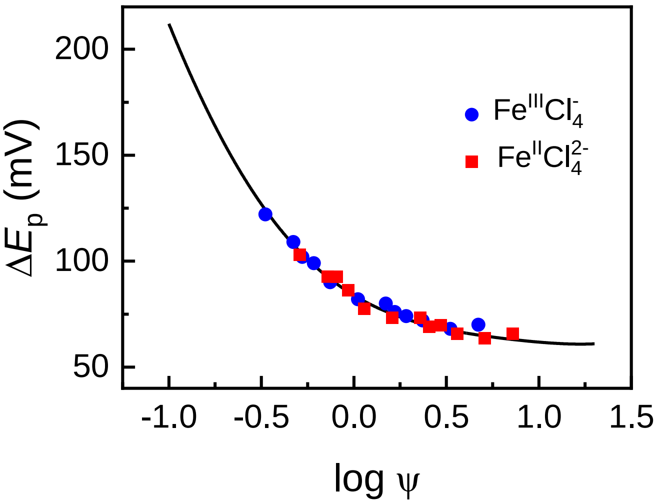

Figure S3. Fitting of experimental data obtained from cyclic voltammetry of $FeCl_4^-$ and $FeCl_4^{2-}$ on a theoretical working curve (solid line) for the determination of $k°$ in DMF + 0.1 M Et_4NBF_4 at T = 25 °C. The dimensionless parameter ψ is defined as:

$$\psi = \frac{(D_{\mathrm{Ox}}/D_{\mathrm{Red}})^{\alpha/2} k°}{\left((nF/RT)\pi D_{\mathrm{Ox}} v\right)^{1/2}}$$

α was assumed to be 0.5, whereas D_{ox} and D_{red} were measured as (8.0 ± 0.8)×10^{-6} cm^2s^{-1} and (2.3 ± 0.1)×10^{-6} cm^2s^{-1}.

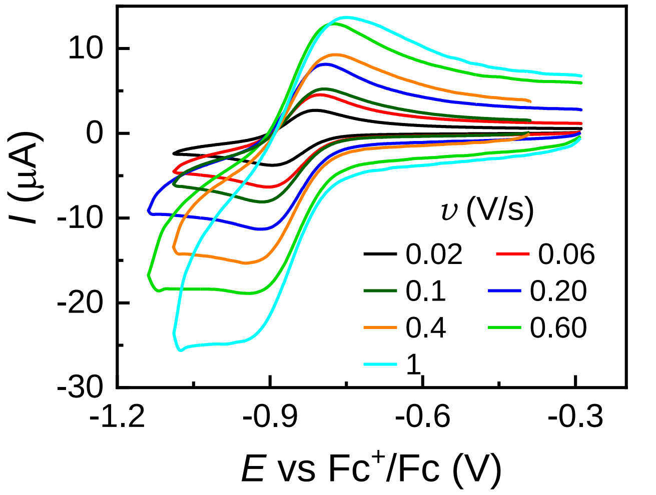

Figure S4. Cyclic voltammetry of 1.0 mM $Fe^{III}L(Cl)$ in DMF + 0.1 M Et_4NBF_4 + 30 mM Et_4NCl, recorded on a GC electrode at different scan rates at T = 25 °C.

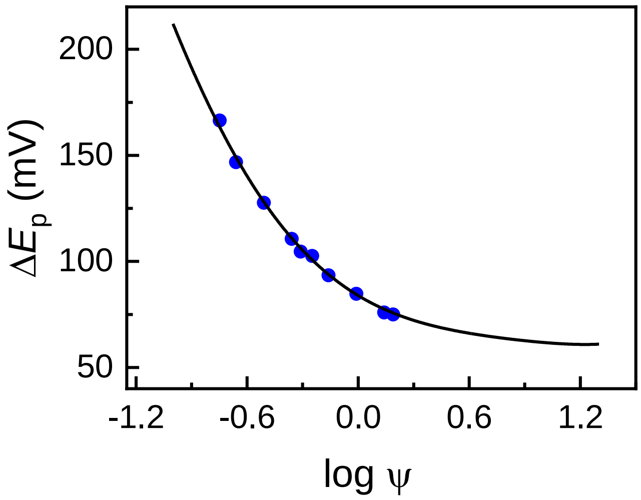

Figure S5. Fitting of experimental data obtained from cyclic voltammetry of $Fe^{III}L(Cl)$ on a theoretical working curve (solid line) for the determination of $k°$ in DMF + 0.1 M Et_4NBF_4 at T = 25 °C. α was assume to be 0.5, whereas the value of D measured for $Fe^{III}L(Cl)$ ((8.0 ± 0.8)×10^{-6} cm^2s^{-1}) was used for both oxidation states of the complex.

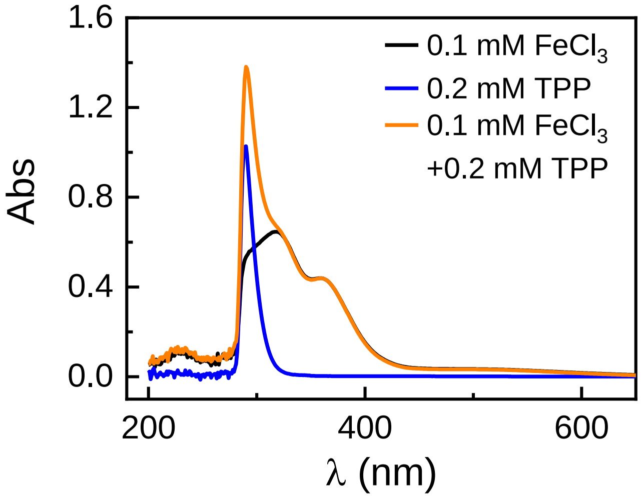

Figure S6. UV-vis spectra of 0.1 mM $FeCl_3$, 0.2 mM TPP and a mixture of 0.1 mM $FeCl_3$ + 0.2 mM TPP, in DMF (path length = 1 cm).

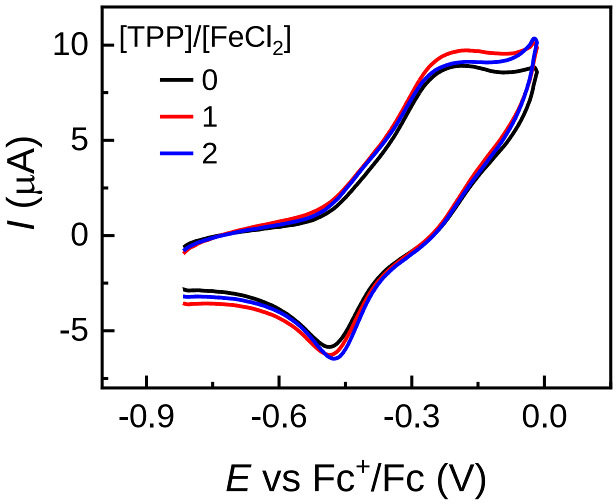

Figure S7. Cyclic voltammetry of 1.08 mM $FeCl_2$ in DMF + 0.1 M Et_4NBF_4, recorded on a GC electrode in the absence and presence of triphenylphosphine (TPP) at v = 0.2 V/s and T = 25 °C.

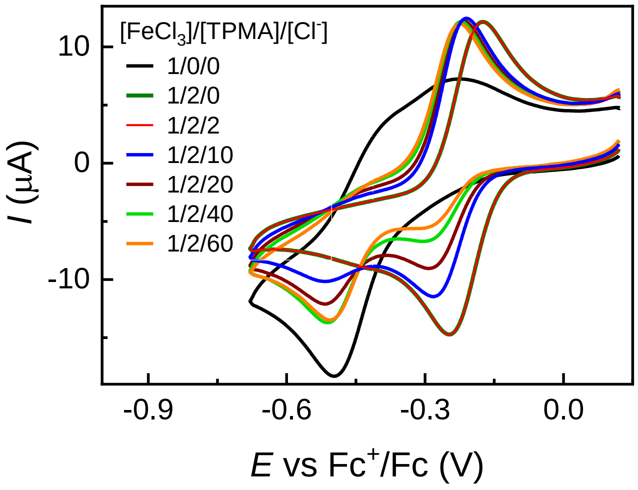

Figure S8. Cyclic voltammetry of 1.1 mM $FeCl_3$ in DMF + 0.1 M Et_4NBF_4 recorded on a GC electrode at v = 0.2 V/s and T = 25 °C, before and after addition of TPMA and Et_4NCl.

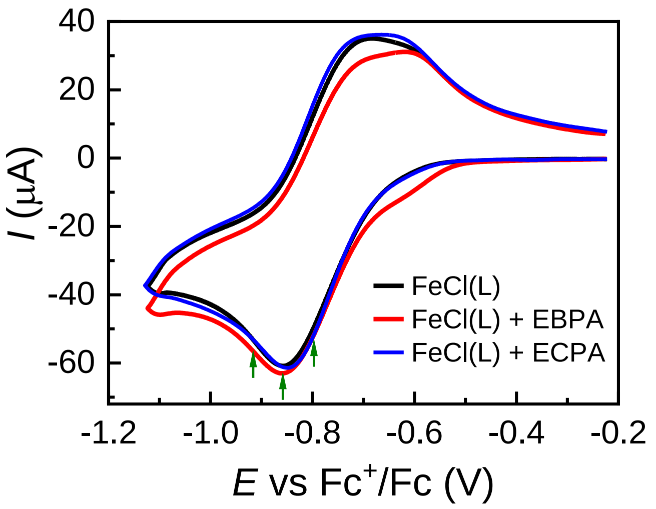

Figure S9. Cyclic voltammetry of 5 mM $Fe^{III}L(Cl)$ in DMF/MMA (50/50, v/v) + 0.1 M Et_4NBF_4 recorded on a GC electrode at v = 0.2 V/s and T = 50 °C, in the absence (black line) and presence of 15 mM ECPA (blue line) or 15 mM EBPA (red line). The arrows indicate E_{app} values used during *e*ATRP: $E_{app} = E_p + 0.06$ V, E_p and $E_p - 0.06$ V.

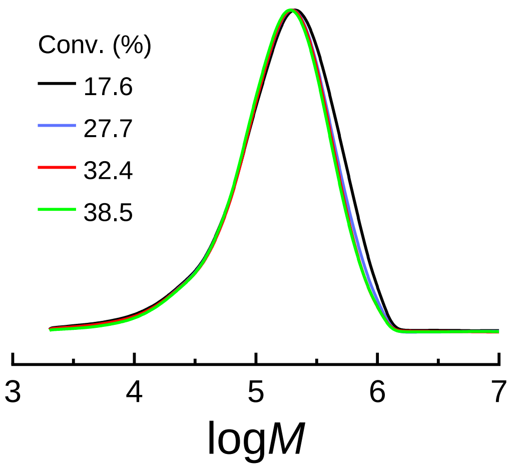

Figure S10. GPC traces of PMMA samples taken at different monomer conversions during *e*ATRP of MMA mediated by $Fe^{III}L(Cl)$ in DMF/MMA (50/50, v/v) + 0.1 M Et_4NBF_4 with EBPA as initiator performed at 50 °C and $E_{app} = E_p + 0.06$ V. Other conditions: [MMA]:[RX]:[$Fe^{III}L(Cl)$] = 100:0.32:0.11, with [$Fe^{III}L(Cl)$] = 5 mM.

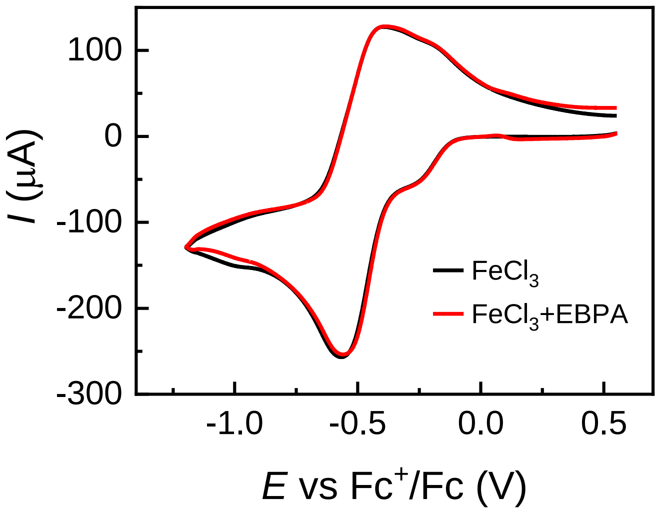

Figure S11. Cyclic voltammetry of 11.75 mM $Fe^{III}Cl_3$ in DMF/MMA (50/50, v/v) + 0.1 M Et_4NBF_4 recorded on a GC electrode at v = 0.2 V/s and T = 55 °C, in the absence (black line) and presence of 47 mM EBPA (red line).

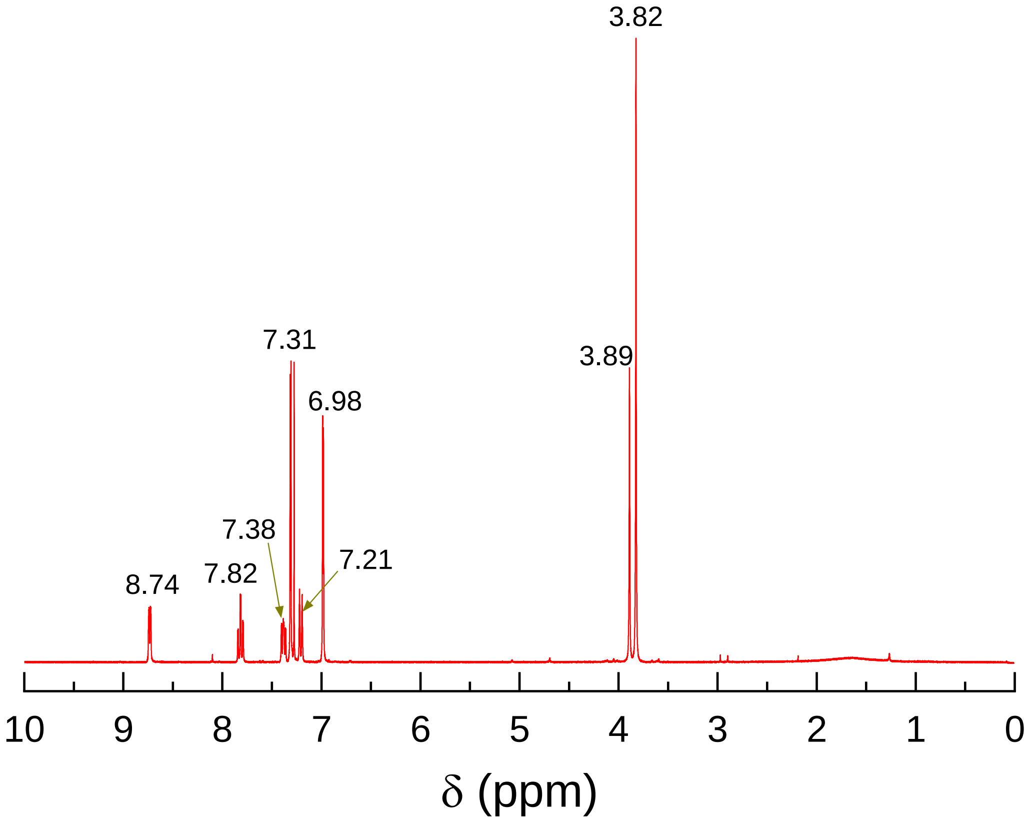

Figure S12. 1H NMR spectrum (300 MHz, $CDCl_3$) of 2-pyridylamino-*N*,*N*-bis(2-methylene-4,6-dichlorophenol.

Table S1. ^{1}H NMR spectral data of 2-pyridylamino-*N*,*N*-bis(2-methylene-4,6-dichlorophenol.

Chemical shift (ppm), multiplicity, number of protons	
This work	Literature[1]
8.74, d, 1H	8.69, d, 1H
7.82, ddd, 1H	7.78, ddd, 1H
7.38, dd, 1H	7.34, dd, 1H
7.31, d, 2H	7.28, s, 2H
7.21, d, 1H	7.16, d, 1H
6.98, d, 2H	6.94, s, 2H
3.89, s, 2H	3.85, s, 2H
3.82, s, 4H	3.79, s, 4H

[1]Reference 1.

Table S2. Elemental analysis of chloro(2-pyridylamino-*N*,*N*-bis(2-methylene-4,6-dichlorophenolate))iron(III).

Element	Predicted (wt%)	Experimental (wt%)
C	43.88	44.46
H	2.58	2.89
N	5.12	4.99

References

1) Reckling, A.M.; Martin, D.; Dawe, L.N.; Decken, A.; Kozak, C.M. Structure and C–C Cross-Coupling Reactivity of Iron(III) Complexes of Halogenated Amine-Bis(Phenolate) Ligands. *J Organomet Chem* **2011**, *696*, 787–794.
